# Host Preference and Performance of the Yellow Peach Moth (*Conogethes punctiferalis*) on Chestnut Cultivars

**DOI:** 10.1371/journal.pone.0157609

**Published:** 2016-06-21

**Authors:** Yanli Du, Jiaxin Zhang, Zengguang Yan, Yongqiang Ma, Mengmeng Yang, Minzhao Zhang, Zhiyong Zhang, Ling Qin, Qingqin Cao

**Affiliations:** 1 Beijing Key Laboratory for Agricultural Application and New Technique, College of Plant Science and Technology, Beijing University of Agriculture, Beijing, People’s Republic of China; 2 State Key Laboratory of Environmental Criteria and Risk Assessment, Chinese Research Academy of Environmental Sciences, Beijing, People’s Republic of China; 3 College of Sciences, China Agricultural University, Beijing, People’s Republic of China; 4 College of Biological science and Engineering, Beijing University of Agriculture, Beijing, People’s Republic of China; Rutgers University, UNITED STATES

## Abstract

Suitability of plant tissues as food for insects varies from plant to plant. In lepidopteran insects, fitness is largely dependent on the host-finding ability of the females. Existing studies have suggested that polyphagous lepidopterans preferentially select certain host plant species for oviposition. However, the mechanisms for host recognition and selection have not been fully elucidated. For the polyphagous yellow peach moth *Conogethes punctiferalis*, we explored the effect of chestnut cultivar on the performance and fitness and addressed the mechanisms of plant-volatile-mediated host recognition. By carrying out laboratory experiments and field investigation on four chestnut *Castanea mollissima* cultivars (Huaihuang, Huaijiu, Yanhong, and Shisheng), we found that *C*. *punctiferalis* females preferentially select Huaijiu for oviposition and infestation, and caterpillars fed on Huaijiu achieved slightly greater fitness than those fed on the other three chestnut cultivars, indicating that Huaijiu was a better suitable host for *C*. *punctiferalis*. Plant volatiles played important roles in host recognition by *C*. *punctiferalis*. All seven chestnut volatile compounds, *α*-pinene, camphene, *β*-thujene, *β*-pinene, eucalyptol, 3-carene, and nonanal, could trigger EAG responses in *C*. *punctiferalis*. The ubiquitous plant terpenoids, *α*-pinene, camphene and *β*-pinene, and their specific combination at concentrations and proportions similar to the emissions from the four chestnut cultivars, was sufficient to elicit host recognition behavior of female *C*. *punctiferalis*. Nonanal and a mixture containing nonanal, that mimicked the emission of *C*. *punctiferalis* infested chestnut fruits, caused avoidance response. The outcome demonstrates the effects of chestnut cultivars on the performance of *C*. *punctiferalis* and reveals the preference-performance relationship between *C*. *punctiferalis* adults and their offspring. The observed olfactory plasticity in the plant-volatile-mediated host recognition may be important for the forming of the relationship between yellow peach moth and chestnuts since it allows the polyphagous herbivores to adjust to variation in volatile emission from their host plants.

## Introduction

Phytophagous insects are generally associated with a particular plant or a constellation of plant species. Insect species that feed on a wide range of plant species belonging to different plant families are called generalists or polyphagous. Even though polyphagous insects can be adaptive to a range of plant species, their fitness differs among various host plants [1]. In lepidopteran insects, many polyphagous species have higher fitness on certain host plants than on others [2]. However, the fitness of lepidopteran insects is largely dependent on the host-finding abilities of the adult females because the hatching larvae (i.e., caterpillars) are often relatively immobile, and their proximity to feeding sites is mostly determined by the judicious choice of the adult female. Adult female lepidopterans have been shown to preferentially select certain host plant species for oviposition [3,4]. A prevailing hypothesis on oviposition preference is that a female will choose those hosts on which caterpillars perform best [5–9]. This preference-performance hypothesis has been clearly supported by a meta-analysis [10]. However, the strength of the relationship between female preference and offspring performance varies among insect groups with different dietary breadth. The preference-performance relationship was stronger in oligophagous insects than in polyphagous or monophagous insects [10]. Even though there was a tremendous body of literature investigating the preference-performance relationship, the effect of dietary breadth on the relationship is still a promising avenue for future research [10]. Especially, the underlying mechanisms of host recognition and selection remain elusive.

For most insect herbivores, olfactory cues are usually used to orientate toward a specific host plant within a plant patch [11]. The most important infochemicals used by herbivores are plant volatiles [2,11–18]. Previous studies on the use of plant volatiles by lepidopteran insects have indicated the existence of preference among potential host plants [19,20]. In some species, the preference of females for some host plants is determined by the concentrations of particular chemical compounds, whereas in other species the preference response is dependent on the relative proportions of ubiquitous plant volatiles [21–24]. It is essential to understand which plant volatiles mediate the insect’s foraging and oviposition behavior. Unfortunately, the mechanisms for host recognition and selection, as well as the linkage between the oviposition preference of females and the fitness of their offspring, have not been fully elucidated. Particularly, few studies have linked the mediation of mothers’ host recognition by plant volatiles with offspring performance. It is therefore necessary to address how plant volatiles affect host recognition by lepidopteran insects and consequently affect offspring performance.

The yellow peach moth, *Conogethes punctiferalis* (Guenée), is widely distributed in tropical and eastern Asia and Australia, and causes damage to many orchard, spice, and vegetable crops [25,26]. Although *C*. *punctiferalis* can feed on a broad range of host plants, its fitness is different on different host plant species [27–29]. A behavioral preference of *C*. *punctiferalis* for some chestnut cultivars has been previously reported [30]. One of the potential mechanisms underlying cultivar selection of *C*. *punctiferalis* is that the adult females can discriminate among volatiles emitted by different chestnut cultivars [31]. However, the ecological implications of volatile-mediated host recognition and selection by *C*. *punctiferalis* remain undetermined, as do the resultant impacts on the fitness of the herbivores. We hypothesized that (1) the suitability of different chestnut cultivars for *C*. *punctiferalis* varies; (2) the adult females can preferentially select certain chestnut cultivars for oviposition; (3) chestnut volatiles play a critical role in the recognition of hosts by *C*. *punctiferalis*; and (4) chestnut-volatile-mediated host recognition may be an ecological adaptation that maximizes the species fitness.

In the present study, experiments were carried out (1) to investigate the field infestation rates of four chestnut cultivars, Huaihuang, Huaijiu, Yanhong, and Shisheng, by *C*. *punctiferalis*; (2) to test the oviposition selection of *C*. *punctiferalis* among the four chestnut cultivars; (3) to test the effects of the chestnut cultivars on the survival, development, and reproduction of *C*. *punctiferalis*; (4) to analyze the volatile emission patterns of the four chestnut cultivars and the temporal changes of volatile emissions in response to an attack of *C*. *punctiferalis*; and (5) to identify the bioactive compounds responsible for attracting or repelling *C*. *punctiferalis*. The major objectives of the study were to address the roles of chestnut volatiles in host recognition by *C*. *punctiferalis* and to determine the relationship between the oviposition preference of females and the fitness of their offspring.

## Materials and Methods

### Ethics statement

We conducted field experiments in an orchard that was authorized by the Huairou Chestnut Germplasm Station of Beijing, China. All experiments carried out in the orchard had been approved by the station, and the field studies did not involve endangered or protected species. The insects used in this study were originated from field collection of caterpillars in cornfields of the Agricultural Experiment Station of Beijing University of Agriculture on October 9th, 2009. The field-captured *C*. *punctiferalis* was a serious pest in China. Therefore, no specific permits were required for the described insect collection and experimentation.

### Plants

Four chestnut (*Castanea mollissima* Blume) cultivars, Huaihuang, Huaijiu, Yanhong, and Shisheng, that were widely grown in local area of Beijing, China, were grown in a 6.7 hm^2^ orchard (40°24'54.54"N, 116°30'20.06"E). Plants used for volatile trapping were 29 years old. The different cultivars were planted in different units of the same orchard, and the space between trees was 2 m while the space between rows was 4 m.

### Insects

A laboratory colony of *C*. *punctiferalis* used in this study had been maintained for approximately 25 generations on maize in climate incubators (RTOP-B, Zhejiang Top Instrument Co., Ltd.) at 23 ± 1°C, RH 75 ± 2%, 16L/8D photoperiod, and 3500 lux light intensity.

### Chemicals

*n*-Hexane (99.9%) was purchased from Thermo Fisher Scientific (China) Co., Ltd. (Shanghai, China), and *α*-pinene (98%) and *β*-pinene (98%) were from J & K Chemical (Shanghai) Ltd. (Shanghai, China). Camphene (78%), eucalyptol (98%), 3-carene (90%), and nonanal (95%) were from TCI (Shanghai) Development Co., Ltd. (Shanghai, China), *β*-thujene was from Chroma Dex (Santa Ana, CA, USA), and *n*-nonyl acetate from Tokyo Kasei Kogyo Co., Ltd. (Toshima, Tokyo, Japan). Other chemicals used in the experiments were all purchased from Sinopharm Chemical Reagent Beijing Co., Ltd. (Beijing, China).

### Investigation of field infestation rate

Infestation of chestnuts by *C*. *punctiferalis* was investigated in the orchard on October 10, 2012. Briefly, five trees of each cultivar were randomly selected, and sampling of chestnut fruits on each tree was conducted at three height levels, i.e., upper, middle, and lower parts of the tree. Five spots at each height level were selected, and three fruits at each spot were collected. The collected fruits were visually examined and then cut open to confirm the infestation status. Forty-five fruits were sampled from each tree, and totally 225 fruits were collected for each cultivar.

### Oviposition selection test

To test the oviposition selection of *C*. *punctiferalis* among the four chestnut cultivars, fruits of Huaihuang, Huaijiu, Yanhong, and Shisheng were simultaneously offered in a wood-frame cage (35 cm × 27 cm × 25 cm) with plastic gauze on side walls to allow the oviposition of *C*. *punctiferalis*. Ten 3-day-old naive (no exposure experience to natural or synthetic sources of chestnut-related volatiles) females and 10 3-day-old males were kept in the cage. A cotton pad soaked with 6–8% honey solution in a 5-ml vial was provided (and renewed every day), and the test was carried out in an environmentally controlled room with temperature 23 ± 3°C, RH 70 ± 5%, 16L:8D photoperiod. For each combination test, one fruit of each cultivar was wrapped with a wet cheese cloth and randomly placed into an inner corner of the cage to allow oviposition of *C*. *punctiferalis* (S1 Fig). The test lasted for 4 days, and during the test the chestnut fruits and the cheesecloth were renewed every day. The renewed fruits were randomly placed in any one of the four cage corners. Ten replicates with a total number of 100 females were performed. Egg numbers on each sheet were counted separately, and the data were statistically treated on the basis of daily average number of eggs by 10 females.

### Development and reproduction test

Laboratory rearing experiments were carried out to determine the performance of *C*. *punctiferalis* on the four chestnut cultivars. In each chestnut a 2-mm hole was punched with a stainless awl, and then neonates of *C*. *punctiferalis* were carefully introduced using a Chinese brush pen onto the nuts. One caterpillar was inoculated for each nut, and ten nuts were placed into a plastic box (15 cm × 9 cm × 8 cm). The boxes were then transferred to a climate incubator to allow the development of the caterpillars under the conditions of 23 ± 1°C, RH 75 ± 2%, 16L/8D photoperiod, and 3500 lux light intensity. Five replicates with a total of 50 caterpillars were performed for each cultivar.

When pupating, the survival of the caterpillars was checked, and newly molted pupae were picked up, counted, weighted and individually placed into a 50-ml tube with a ventilated lid, and then transferred to the incubator to allow emergence of the moths. Adult moths developed from the larvae fed on the same chestnut cultivar were pooled and then transferred two by two (one male *vs* one female) into cylindrical plastic-mesh cages (35 cm × 27 cm × 25 cm). The moths were provided with 6–8% honey solution in a cotton pad in a 5-ml vial, and the honey solution was renewed every day. An apple wrapped with wet cheesecloth was placed into the cage, and the female was allowed to lay eggs (F1) on it. The cheesecloth was renewed every day until the death of the female moth. The eggs were counted and then placed into a plastic box (15 cm × 9 cm × 8 cm) to allow hatching under experimental conditions. The hatched F1 caterpillars were collected and counted every day until no neonates were available anymore. Ten repetitions with a total number of 20 moths (10 males and 10 females, respectively) were performed for each group of moths with experience on the same chestnut cultivar. The developmental duration of eggs and caterpillars, as well as the survival rate of caterpillars and the fecundity of adult females (number of F1 eggs per female), were determined using the methods described in our previous study [28].

### Collection and identification of chestnut volatiles

Chestnut volatiles were collected from August 18 to 28, 2012, during which time the chestnut fruits were in the enlargement stage. Meanwhile, in this period the *C*. *punctiferalis* adults of the second generation were transferring from peach to chestnut [32]. Chestnut volatiles were collected for fruits of all four cultivars. Briefly, a chestnut twig (ca. 15 cm long) with three fruits on it was wrapped in a 48.2 cm × 59.6 cm Reynolds oven bag (Reynolds Kitchens, Richmond, VA, USA), and the bag was tightened with a twist tie around the stem of the twig (S2 Fig). In the upper corners of the bag, two small holes were made; one was connected to a Teflon tube and the other was connected to a glass tube (6 mm diameter, 10 cm long) containing 50 mg of Porapark Q adsorbent (80–100 mesh, Waters Corporation, Milford, MA, USA). Humidified and purified air was pushed at a rate of 450 ml/min by a QC-1S pump (Labour Protection Science Research Institute of Beijing, Beijing, China) into the bag through the Teflon tube and was pulled out through the glass tube by another QC-1S pump. Volatiles were trapped by the Porapark Q adsorbent when they passed through the glass tube. Four setups were used in parallel for the collection of volatiles, and one setup made in the same way but containing no twig was used as a control (blank). The collection was run for 4 h, and three replicates were performed for each cultivar.

To investigate time-dependent emission of chestnut volatiles in response to *C*. *punctiferalis* infestation, volatiles were collected from Huaijiu fruits after being infested by the caterpillars for 12, 36, and 60 h. In detail, two third-instar caterpillars were introduced onto each of the three fruits on the same twig (~15 cm long) at approximately 19:00 PM (S3 Fig). The caterpillars were starved for 12 h before being placed onto the fruits and normally bored into the fruits within 3 h after coming into contact with them. Starting the next day, volatiles emitted from the infested fruits were collected from 7:00 to 11:00 AM on days 1, 2 and 3 following the methods described above. Three replicates were performed.

After collection, the trapped volatiles were eluted from the adsorbent using 350 μl of chromatography-grade *n*-hexane (99.9%) and then stored at -20°C until use for analysis. For analysis, 200 μl of eluent was subsampled and 1 μl of *n*-nonyl acetate (42 ng/μl) was added as an internal standard (IS). Characterization and quantification of chestnut volatiles were carried out using an Agilent 6890 gas chromatograph (GC) coupled to an Agilent 5975 Mass Spectrometer (MS). The GC was equipped with a HP-5MS column (30 m × 0.25 mm × 0.25 μm). Helium was used as carrier gas with a constant flow of 26 cm/s. The injector temperature was 210°C and the GC-MS transfer line temperature was 280°C, source 230°C, quadrupole 150°C, ionization potential 70 eV, and scan range 30–300 m/z. One microliter of sample was injected by splitless mode. Following injection, the column temperature was maintained at 55°C for 1 min and then programmed at 5°C/min to 200°C (held for 5 min). Compounds were identified by comparing mass spectra with NIST library spectra (Agilent Technologies, Palo Alto, CA, USA), and some of them (*α*-pinene, camphene, *β*-thujene, *β*-pinene, eucalyptol, 3-carene, and nonanal) were confirmed with authentic reference compounds. Compounds were quantified by their total ion abundance relative to that of the internal standard.

### Behavioral assay

A Y-tube olfactometer was used to test dual choice responses of *C*. *punctiferalis* to single volatiles and an array of blends. The olfactometer consisted of two glass chambers (2.5 cm diameter, 10 cm long) each coupled to one of two 10-cm-long arms (2.5 cm diameter) of the Y-tube olfactometer that converge into a 13-cm-long common arm (2.5 cm diameter). Seven single compounds, *α*-pinene, camphene, *β*-thujene, *β*-pinene, eucalyptol, 3-carene, and nonanal, as well as the simulated blends S-HH-IT, S-HJ-IT, S-YH-IT, S-SS-IT, and S-HJ-IF60 (Table 1) mimicking the emissions of chestnut fruits were tested for the behavioral responses of *C*. *punctiferalis* with mineral oil as a control. Test solutions used for the behavioral assay were made by serially dissolving each single compound or volatile mixture into laboratory-grade mineral oil to obtain the same concentration as in the natural Huaijiu blend (Tables 2 and 3). The preparation of the simulated blends followed the prescriptions presented in Table 1, in which the combinations of compounds were based on the concentrations and proportions quantified in the natural blends (Tables 2 and 3). Afterwards, the test solution (10 μl) was applied onto a 1 cm × 5 cm filter paper, which was then placed into one of the chambers of the olfactometer, and another filter paper loaded with 10 μl mineral oil was placed into the other chamber to serve as a control. Moistened and charcoal-filtered air was pumped into each of the two chambers at a rate of 500 ml/min controlled by flow meters (LZYIA Instrument Co. Ltd, China). One moth was introduced into the entrance of the common arm of the Y-tube olfactometer using a glass vial, and its behavioral response was observed under a 25-W red light lamp. The test for each moth lasted 3 min, and the behavioral response was classified as a choice if the test moth passed over 1/3 the length of the Y-tube arm associated with either chemical or mineral oil and stayed there for more than 1 min. Conversely, no-choice was assigned if the test moth remained in the common arm for 3 min. A new pair of filter paper was used for each individual female tested, and the position of the lateral chambers along with the Y-tube olfactometer was systematically exchanged after testing 2 moths to avoid positional bias. The Y-tube olfactometer was flushed with clean water and alcohol (75%) after the testing of 10 moths and then air dried before being used for the next test. Each individual moth was used only once, and the moths were allowed to acclimatize to the test conditions for 1 h before the start of the test. All assays were conducted between 19:00 and 23:00 at temperature 25 ± 3°C and RH 60 ± 5%. At least 40 individual moths were used for each dual choice test, and both virgin and mated females (3 day old) were tested.

**Table 1:** Quantity and proportions of chestnut volatiles in simulated blends used for behavioral and EAG assay*.

Compound	S-HH-IT		S-HJ-IT		S-YH-IT		S-SS-IT		S-HJ-IF60	
	Quantity (ng/ml)	Proportion (%)	Quantity (ng/ml)	Proportion (%)	Quantity(ng/ml)	Proportion (%)	Quantity (ng/ml)	Proportion (%)	Quantity (ng/ml)	Proportion (%)
α-Pinene	156	25.3	116	27.6	607	22.5	248	24.7	500	21.6
camphene	54	8.8	39	9.2	141	5.2	80	7.9	182	7.8
β-thujene	248	40.2	156	37.2	1300	48.3	411	40.9	874	37.8
β-pinene	158	25.7	109	26.0	645	24.0	266	26.5	493	21.3
eucalyptol	—	—	—	—	—	—	—	—	108	4.7
3-carene	—	—	—	—	—	—	—	—	46	2.0
nonanal	—	—	—	—	—	—	—	—	112	4.8

* S-HH-IT = blend simulated intact Huaihuang fruits

S-HJ-IT = blend simulated intact Huaijiu fruits, S-YH-IT = blend simulated intact Yanhong fruits, S-SS-IT = blend simulated intact Shisheng fruits, S-HJ-IF60 = blend simulated Huaijiu fruits infested by *C*. *punctiferalis* for 60 h.

**Table 2:** Compounds emitted from intact fruits of four chestnut cultivars, Huaihuang, Huaijiu, Yanhong, and Shisheng*.

Number	Compound	Amount (ng/nut/h)				Proportion (%)			
		HH-IT	HJ-IT	YH-IT	SS-IT	HH-IT	HJ-IT	YH-IT	SS-IT
1	*α*-pinene	1.56±0.02^b^	1.16±0.01^a^	6.07±0.16^d^	2.48±0.07^c^	25.38±1.06^b^	27.66±0.20^b^	18.49±0.09^a^	20.76±0.38^a^
2	camphene	0.54±0.01^b^	0.39±0.00^a^	1.41±0.04^d^	0.80±0.02^c^	8.73±0.05^c^	9.18±0.05^c^	4.28±0.04^a^	6.67±0.24^b^
3	*β*-thujene	2.48±0.12^a^	1.56±0.03^a^	13.00±0.46^c^	4.11±0.14^b^	40.18±1.06^b^	37.27±0.83^a^^b^	39.59±0.26^b^	34.31±0.81^a^
4	*β*-pinene	1.58±0.04^b^	1.09±0.03^a^	6.45±0.15^d^	2.66±0.03^c^	25.70±0.09^c^	25.89±0.68^c^	19.65±0.12^a^	22.22±0.57^b^
6	*β*-ocimene	—	—	2.36±0.06	—	—	—	7.20±0.03	—
9	(*E*)-2-butenoic acid,2-(methylene- cyclopropyl)prop-2-yl ester	—	—	3.54±0.09^b^	1.92±0.02^a^	—	—	10.77±0.03^a^	16.04±0.37^b^
Total		6.16±0.15^a^	4.19±0.01^a^	32.82±0.95^c^	11.96±0.14^b^	—	—	—	—

* Values are mean ± SE (*n* = 3).

HH-IT = intact Huaihuang fruits, HJ-IT = intact Huaijiu fruits, YH-IT = intact Yanhong fruits, SS-IT = intact Shisheng fruits.

^a-d^ Different letters in the same row indicate a significant difference (Tukey-HSD test after ANOVA, *P* < 0.05).

**Table 3:** Compounds emitted from intact and *Conogethes punctiferalis* infested Huaijiu fruits*.

Number	compound	Amount (ng/nut/h)				Proportion (%)			
		HJ-IT	HJ-IF12	HJ-IF36	HJ-IF60	HJ-IT	HJ-IF12	HJ-IF36	HJ-IF60
1	*α*-pinene	1.16±0.01^a^	3.99±0.16^b^	4.64±0.09^b^^c^	5.00±0.25^c^	27.66±0.20^d^	14.74±0.33^b^	8.50±0.21^a^	17.32±0.32^c^
2	camphene	0.39±0.00^a^	1.85±0.06^c^	1.48±0.04^b^	1.82±0.04^c^	9.18±0.05^d^	6.83±0.02^c^	2.71±0.05^a^	6.31±0.08^b^
3	*β*-thujene	1.56±0.03^a^	8.77±0.34^b^	10.56±0.43^c^	8.74±0.06^b^	37.26±0.83^c^	32.41±0.39^b^	19.34±0.49^a^	30.35±0.80^b^
4	*β*-pinene	1.09±0.03^a^	4.46±0.13^b^	5.48±0.11^c^	4.93±0.25^b^^c^	25.89±0.68^c^	16.51±0.20^b^	10.05±0.14^a^	17.07±0.30^b^
5	eucalyptol	—	—	—	1.08±0.04	—	—	—	3.75±0.01
6	*β*-ocimene	—	2.98±0.12^b^	26.32±0.66^c^	0.72±0.00^a^	—	11.00±0.03^b^	48.23±0.19^c^	2.50±0.06^a^
7	3-carene	—	—	—	0.46±0.02	—	—	—	1.60±0.01
8	nonanal	—	—	—	1.12±0.04	—	—	—	3.88±0.01
9	(*E*)-2-butenoic acid,2-(methylene- cyclopropyl)prop-2-yl ester	—	5.01±0.28^a^	6.09±0.03^b^	4.98±0.27^a^	—	18.50±0.31^c^	11.16±0.19 ^a^	17.22±0.35^b^
Total		4.19±0.01^a^	27.06±1.05^b^	54.57±1.17^c^	28.85±0.97^b^	—	—	—	—

* Values are mean ± SE (*n* = 3).

HJ-IT = intact Huaijiu fruits, HJ-IF12 = Huaijiu fruits infested by *Conogethes punctiferalis* for 12 h, HJ-IF36 = Huaijiu fruits infested by *C*. *punctiferalis* for 36 h, HJ-IF60 = Huaijiu fruits infested by *C*. *punctiferalis* for 60 h.

^a-c^ Different letters in the same row indicate significant difference (Tukey-HSD test after ANOVA, *P* < 0.05).

### Electroantennogram assay

The sensory responses of *C*. *punctiferalis* to chestnut volatiles were evaluated by measuring electroantennogram (EAG) responses. In brief, the antenna was cut off from the head of a 3-day-old female moth and suspended between 2 electrodes using a Spectra 360 electrode gel (Parker Laboratories Inc., Fairfield, NJ, USA). The electrodes were connected to a high-impedance DC amplifier (Syntech UN-06, Syntech^®^, Hilversum, Netherlands). The mounted antenna was exposed to an air stream carrying the test compounds puffed away from a steel tube. A test solution aliquot of 10 μl was pipetted onto a piece of filter paper (5 mm × 50 mm), which was immediately inserted into a glass Pasteur pipette (6 mm diameter, 10 cm length). After loading the filter paper, the pipette was sealed with parafilm at the two ends until it was used for EAG testing (within 1 h). The narrow end of the Pasteur pipette was inserted through a small hole in the wall of the steel tube. Antennae were positioned approximately 10 mm from the outlet of the steel tube. For stimulation, 0.5 ml of purified charcoal-filtered air was led through the Pasteur pipette for 0.3 s into the main air stream running through the steel tube (150 ml/min). An electrically controlled air flow controller (Syntech CS-05, Syntech^®^, Hilversum, Netherlands) triggered by manual operation was used for the stimulation. At least 60 s was allowed between successive stimulations, and the stimulations with different volatiles were made in random order. Ten microliters of mineral oil and *n*-hexanol were used as control and standard stimuli, respectively. In each test series of volatiles, the control and standard stimuli were applied subsequently after four successive stimulations. Normalization was achieved by dividing the peak EAG amplitude of the test puff with the average EAG amplitude of the two nearest standard stimulations after subtracting the amplitude recorded in response to the mineral oil. Both virgin and mated females (3 day old) were tested, and each volatile or blend was tested on 15 antennae.

### Statistical analysis

The Tukey-HSD test after ANOVA was used to determine the significance of differences (*P* = 0.05) in development and reproduction of *C*. *punctiferalis*. Additionally, differences between chestnut cultivars in oviposition selection, field infestation rate, and the amount of volatile emissions, as well as EAG responses among different volatiles were also evaluated using the Tukey-HSD test after ANOVA. A non-parametric procedure called *Chi*-squared analysis was performed to test the significance of differences between numbers of moths that made a choice between mineral oil (control) and the chestnut volatiles (a 50:50 probability was set between the numbers of moths choosing either side of the Y-tube). Moths that were scored as no choice were excluded from the statistical analysis. All statistics were performed using the SPSS16.0 statistical software.

## Results

### Infestation rate

**Fig 1:**
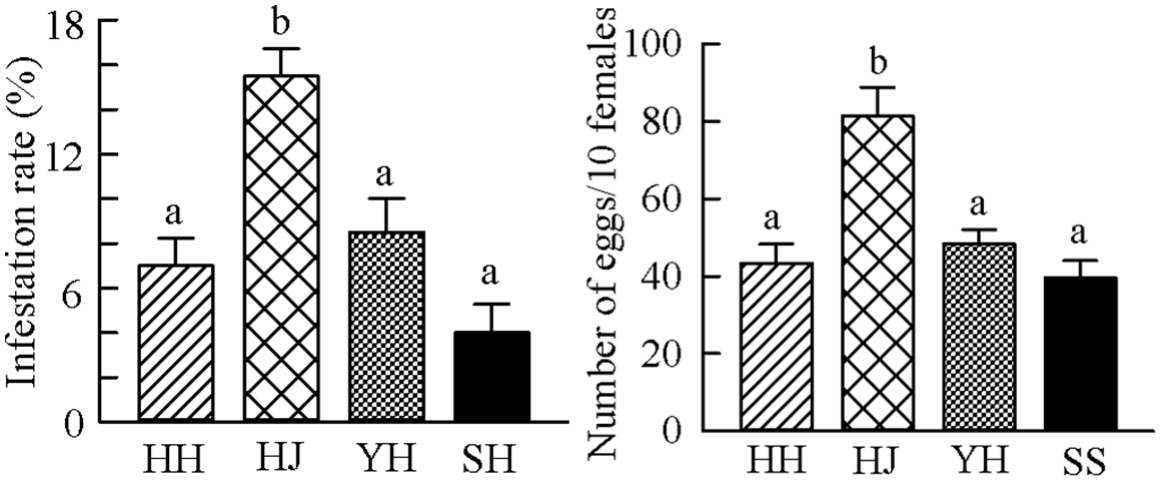
Field infestation rate and oviposition choice of *Conogethes punctiferalis* on four chestnut cultivars. HH = Huaihuang, HJ = Huaijiu, YH = Yanhong, SS = Shisheng. (a) Field infestation rate of four chestnut cultivars by *C*. *punctiferalis*. Bars represent means ± SE (*n* = 5). Different letters on the bars indicate significant differences among the four chestnut cultivars (Tukey-HSD test after ANOVA, *P* < 0.05), (b) Oviposition choice of *C*. *punctiferalis* among four chestnut cultivars. Bars represent means ± SE (*n* = 10) of the daily laying eggs per 10 females. Different letters on the bars indicate significant difference among the four chestnut cultivars (Tukey-HSD test after ANOVA, *P* < 0.05).

Field infestation rates differed significantly among chestnut cultivars (Tukey-HSD test after ANOVA, *F*(3, 16) = 13.818, *P* < 0.001; Fig 1a). The highest infestation rate (15.5%) was in Huaijiu cultivar, followed by Yanhong (8.5%), Huaihuang (7.0%), and Shisheng (4.0%) cultivars.

### Oviposition selection

Among the four chestnut cultivars simultaneously offered, adult female *C*. *punctiferalis* chose to lay more eggs on Huaijiu fruits than on the fruits of the other three cultivars (Tukey-HSD test after ANOVA, *F*(3, 36) = 13.437, *P* < 0.001; Fig 1b). The daily average number of eggs per 10 females was 81.5 in Huaijiu, followed by Yanhong (48.4), Huaihuang (43.4), and Shisheng (39.7). The number of eggs on Huaijiu was significantly larger than the numbers on Yanhong, Huaihuang, and Shisheng, indicating an oviposition preference of *C*. *punctiferalis* for Huaijiu cultivar.

### Development and reproduction

**Fig 2:**
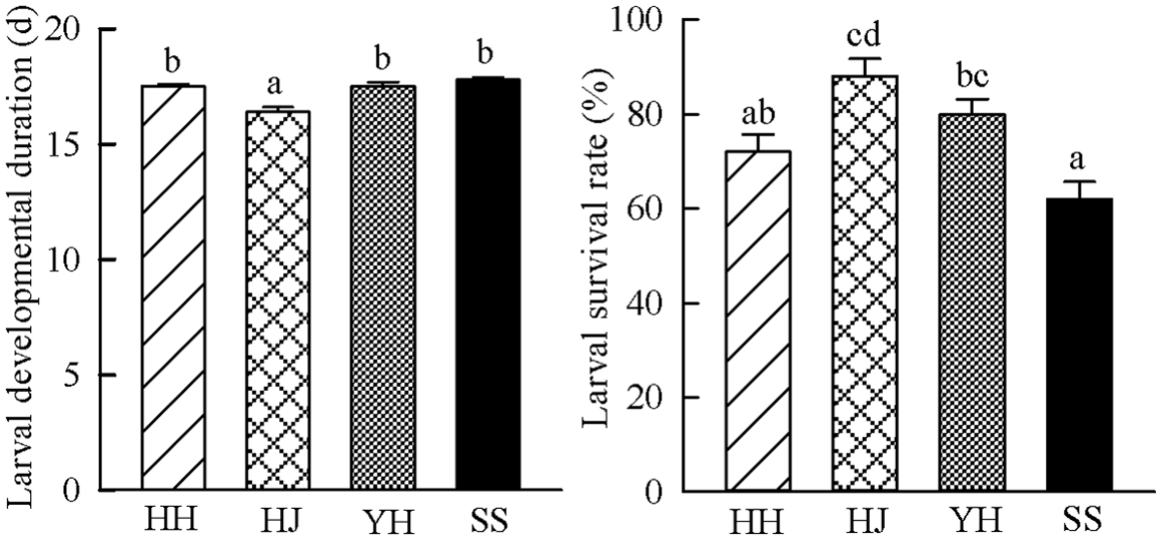
Developmental duration and survival rate of *Conogethes punctiferalis* caterpillars on four chestnut cultivars. HH = Huaihuang, HJ = Huaijiu, YH = Yanhong, SS = Shisheng. (a) Larval developmental duration of *Conogethes punctiferalis* on four chestnut cultivars, (b) Larval survival rate of *Conogethes punctiferalis* on the four chestnut cultivars. Bars represent means ± SE. Significant differences among the four chestnut cultivars are indicated by different letters on each bar (Tukey-HSD test after ANOVA, *P* < 0.05).

**Fig 3:**
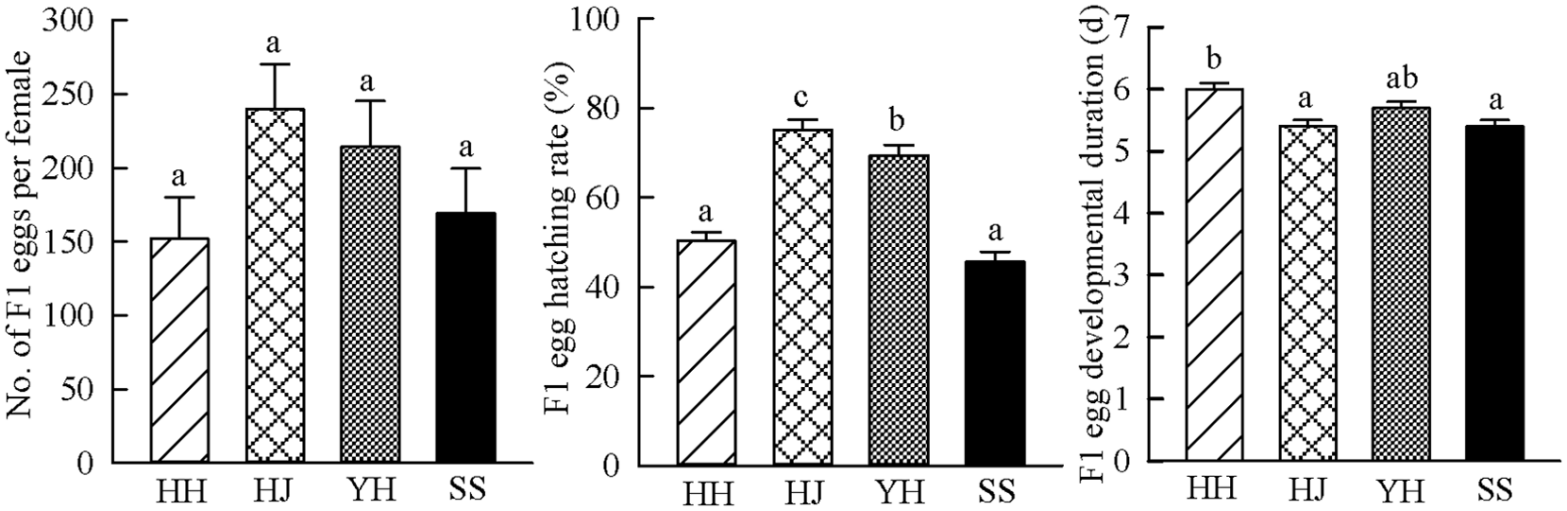
Reproduction and F1 egg development of *Conogethes punctiferalis* on four chestnut cultivars. HH = Huaihuang, HJ = Huaijiu, YH = Yanhong, SS = Shisheng. (a) Number of F1 eggs per female *Conogethes punctiferalis*, (b) F1 egg hatching rate of *Conogethes punctiferalis*, (c) F1 egg developmental duration of *Conogethes punctiferalis*. Bars represent means ± SE. Significant differences among four cultivars are indicated by different letters on each bar (Tukey-HSD test after ANOVA, *P* < 0.05).

Chestnut cultivar had significant impacts on larval developmental duration and survival (Fig 2). The caterpillars fed on Huaijiu had significantly faster growth rate than those fed on Huaihuang, Yanhong or Shisheng (Tukey-HSD test after ANOVA, *F*(3, 147) = 14.739, *P* < 0.001; Fig 2a). Additionally, the caterpillars fed on Huaijiu had a higher survival rate than those fed on Huaihuang and Shisheng (Tukey-HSD test after ANOVA, *F*(3, 16) = 9.513, *P* = 0.001; Fig 2b), and also had slight but not significant higher survival rate than those fed on Yanhong, indicating that the caterpillars fed on Huaijiu had better performance. Rearing of *C*. *punctiferalis* on different chestnut cultivars also impacted the performance of the adults. The number of F1 eggs produced by each adult female decreased with experience from Huaijiu to Yanhong to Shisheng to Huaihuang, although the differences were not statistically significant (Tukey-HSD test after ANOVA, *F*(3, 36) = 1.806, *P* = 0.164; Fig 3a). Furthermore, the effects of the chestnut cultivars on the performance of *C*. *punctiferalis* could be observed in the F1 generation. F1 eggs with parents’ experience on Huaijiu had higher hatching rate (75.2%) than those with parent’s experience on Yanhong (69.5%), Huaihuang (50.4%) or Shisheng (45.6%) (Tukey-HSD test after ANOVA, *F*(3, 36) = 129.933, *P* < 0.001; Fig 3b). Moreover, F1 eggs with parents reared on Huaijiu had shorter developmental duration than those with parents reared on Huaihuang (Tukey-HSD test after ANOVA, *F*(3, 36) = 8.348, *P* < 0.001; Fig 3c), and also had slight but not significant shorter developmental duration than those with parents reared on Yanhong and Shisheng (Fig 3c). Altogether, these data demonstrate that *C*. *punctiferalis* reared on Huaijiu had better performance than on Huaihuang, Yanhong or Shisheng in terms of the favored aspects of the selected biological parameters.

### Chestnut volatiles

**Fig 4:**
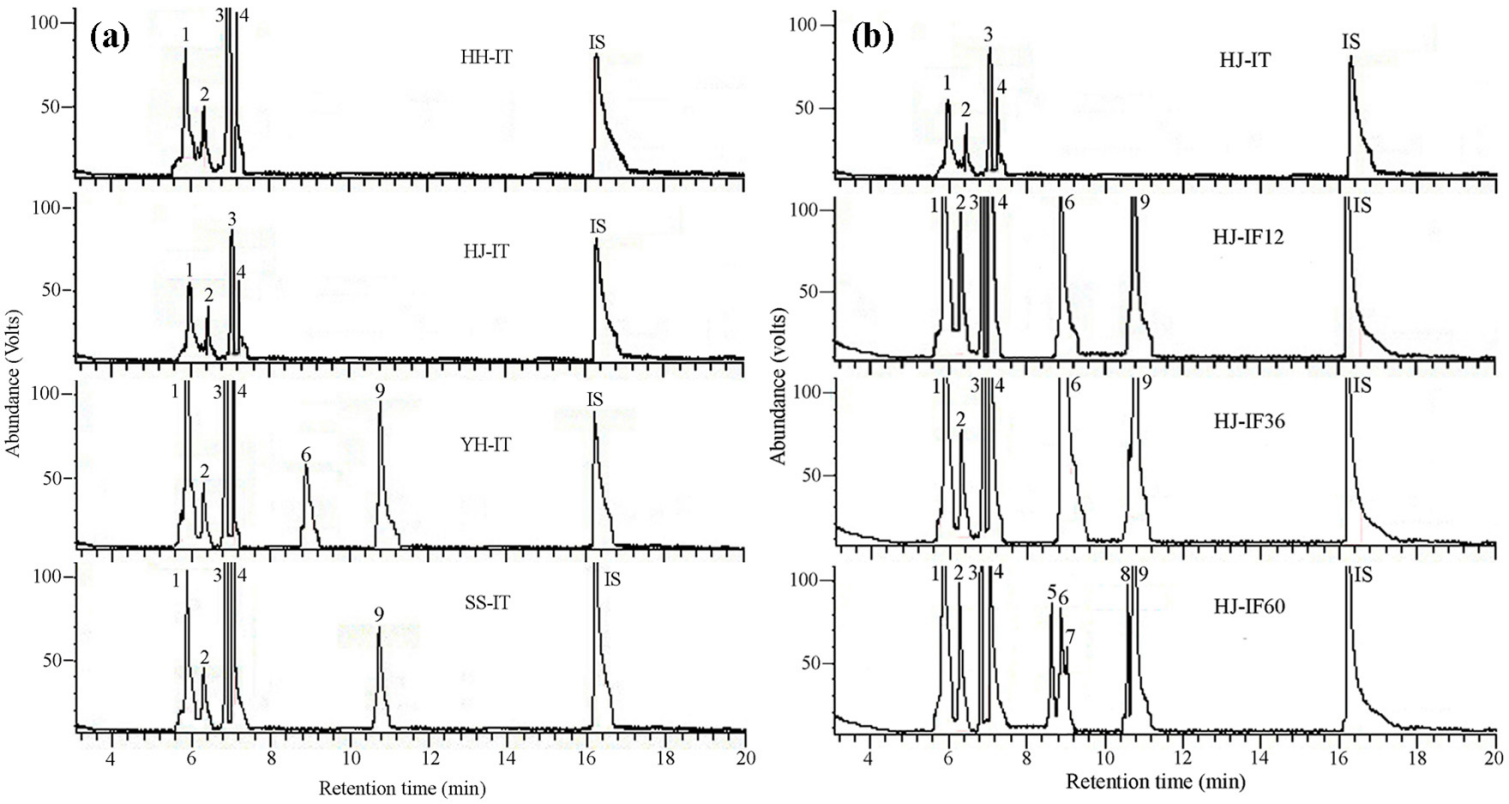
Representative total ion current chromatograms of the volatiles emitted by intact and *Conogethes punctiferalis* infested Huaijiu fruits. (a) Representative total ion current chromatograms of the volatiles emitted by intact fruits of four chestnut cultivars, HH-IT = intact Huaihuang fruits, HJ-IT = intact Huaijiu fruits, YH-IT = intact Yanhong fruits, SS-IT = intact Shisheng fruits, (b) Representative total ion current chromatograms of the volatiles emitted by intact and *C*. *punctiferalis* infested Huaijiu fruits, HJ-IT = intact Huaijiu fruits, HJ-IF12 = Huaijiu fruits infested by *C*. *punctiferalis* for 12 h, HJ-IF36 = Huaijiu fruits infested by *C*. *punctiferalis* for 36 h, HJ-IF60 = Huaijiu fruits infested by *C*. *punctiferalis* for 60 h. (1) *α*-pinene, (2) camphene, (3) *β*-thujene, (4) *β*-pinene, (5) eucalyptol, (6) *β*-ocimene, (7) 3-carene, (8) nonanal, (9) (*E*)-2-butenoic acid,2-(methylene-cyclopropyl)prop-2-yl ester, (IS) internal standard (*n*-nonyl acetate).

Volatiles emitted from intact fruits of the four chestnut cultivars and from *C*. *punctiferalis*-infested Huaijiu fruits were collected and analyzed. Representative total ion current chromatograms of the volatiles emitted from intact and *C*. *punctiferalis*-infested fruits are presented in Fig 4. The average amount as well as the proportion of each volatile is presented in Tables 2 and 3.

In total, 6 volatile compounds were emitted from the intact fruits of the four chestnut cultivars (Fig 4a). Four terpenoids, *α*-pinene, camphene, *β*-thujene and *β*-pinene, were emitted by all four chestnut cultivars, whereas *β*-ocimene and (*E*)-2-butenoic acid, 2-(methylene-cyclopropyl)prop-2-yl ester were only released by Yanhong or Shisheng. Additionally, there was an overall difference in the total amount of volatile emissions among the four chestnut cultivars (Tukey-HSD test after ANOVA, *F*(3, 8) = 725.121, *P* < 0.001; Table 2). The total amount of volatiles was in the order of Yanhong > Shisheng > Huaihuang > Huaijiu. The proportions of *α*-pinene, camphene, and *β*-pinene were high in the total emissions of Huaijiu, whereas the proportion of *β*-thujene was high in Huaihuang.

A total of 9 volatiles were emitted from *C*. *punctiferalis*-infested Huaijiu fruits (Fig 4b). The four terpenoids (*α*-pinene, camphene, *β*-thujene and *β*-pinene) emitted by intact Huaijiu fruits (HJ-IT) were also emitted by infested ones. *β*-ocimene and (*E*)-2-butenoic acid, 2-(methylene-cyclopropyl)prop-2-yl ester were released from fruits infested for 12 h (HJ-IF12), 36 h (HJ-IF36) and 60 h (HJ-IF60), while eucalyptol, 3-carene, and nonanal were specifically released by fruits after being infested for 60 h (HJ-IF60), indicating time-dependent patterns of volatile emissions in response to the attack of *C*. *punctiferalis*. Consistently, fruits infested by *C*. *punctiferalis* released much larger amounts of volatiles than intact fruits (Table 3), and the largest emissions were observed from fruits having been infested for 36 h.

### Behavioral responses

**Fig 5:**
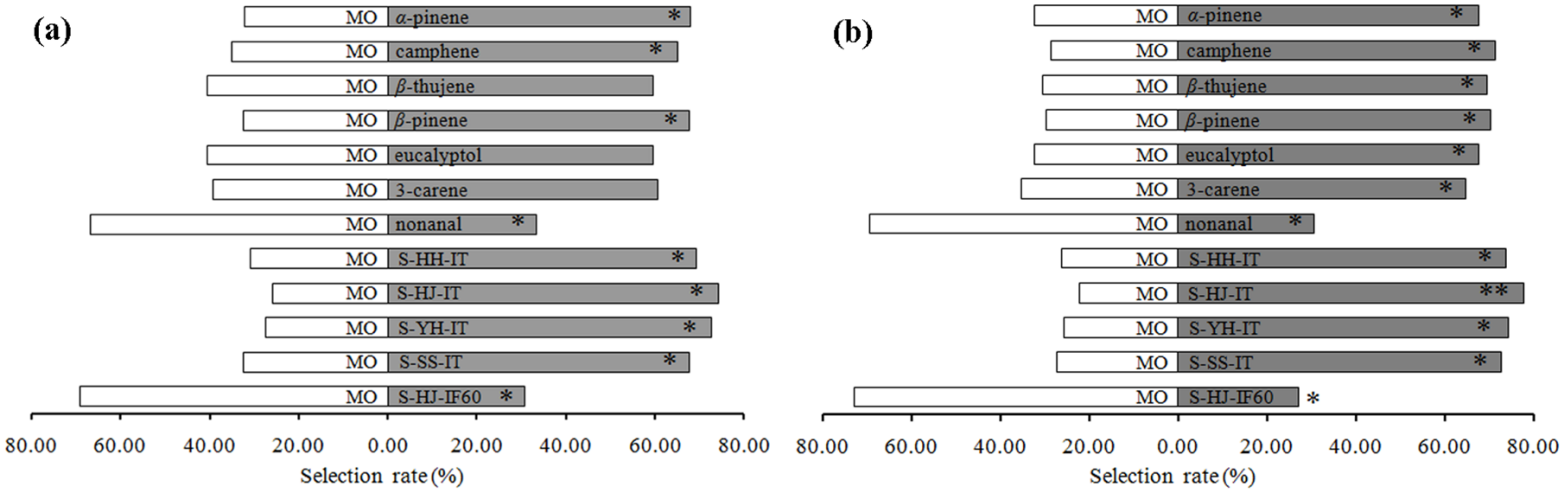
Choice distribution of female *Conogethes punctiferalis* for chestnut volatiles in dual choice assay in a Y tube. (a) Selection rates of virgin females for chestnut volatiles in dual choice assay with mineral oil as control, (b) Selection rates of mated females for chestnut volatiles with mineral oil as control, MO = mineral oil, S-HH-IT = simulated blend mimicking the emission of intact Huaihuang fruits, S-HJ-IT = simulated blend mimicking the emission of intact Huaijiu fruits, S-YH-IT = simulated blend mimicking the emission of intact Yanhong fruits, S-SS-IT = simulated blend mimicking the emission of intact Shisheng fruits, S-HJ-IF60 = simulated blend mimicking the emission of Huaijiu fruits infested by *Conogethes punctiferalis* for 60 h. Stars indicate significant difference within a choice test using *x*^2^ test (*P < 0.05, **P < 0.001).

The behavioral responses of virgin and mated *C*. *punctiferalis* females to seven single compounds and a range of artificially prepared volatile blends (Table 1) mimicking the emissions of chestnut fruits were investigated with mineral oil as a control. Virgin female moths were significantly attracted to *α*-pinene, camphene, *β*-pinene, and mixture blends simulating the emissions of Huaihuang (S-HH-IT), Huaijiu (S-HJ-IT), Yanhong (S-YH-IT), and Shisheng (S-SS-IT) intact fruits (Fig 5a). However, avoidance responses to nonanal and a mixture blend simulating the emissions of *C*. *punctiferalis*-infested fruits (S-HJ-IF60) were observed. After mating, female moths showed preference for *α*-pinene, camphene, *β*-thujene, *β*-pinene, eucalyptol and 3-carene, as well as the mixture blends S-HH-IT, S-HJ-IT, S-YH-IT, and S-SS-IT. Similar to virgin females, mated females also showed significant avoidance responses to nonanal and the mixture blend S-HJ-IF60 (Fig 5b).

### EAG responses

**Fig 6:**
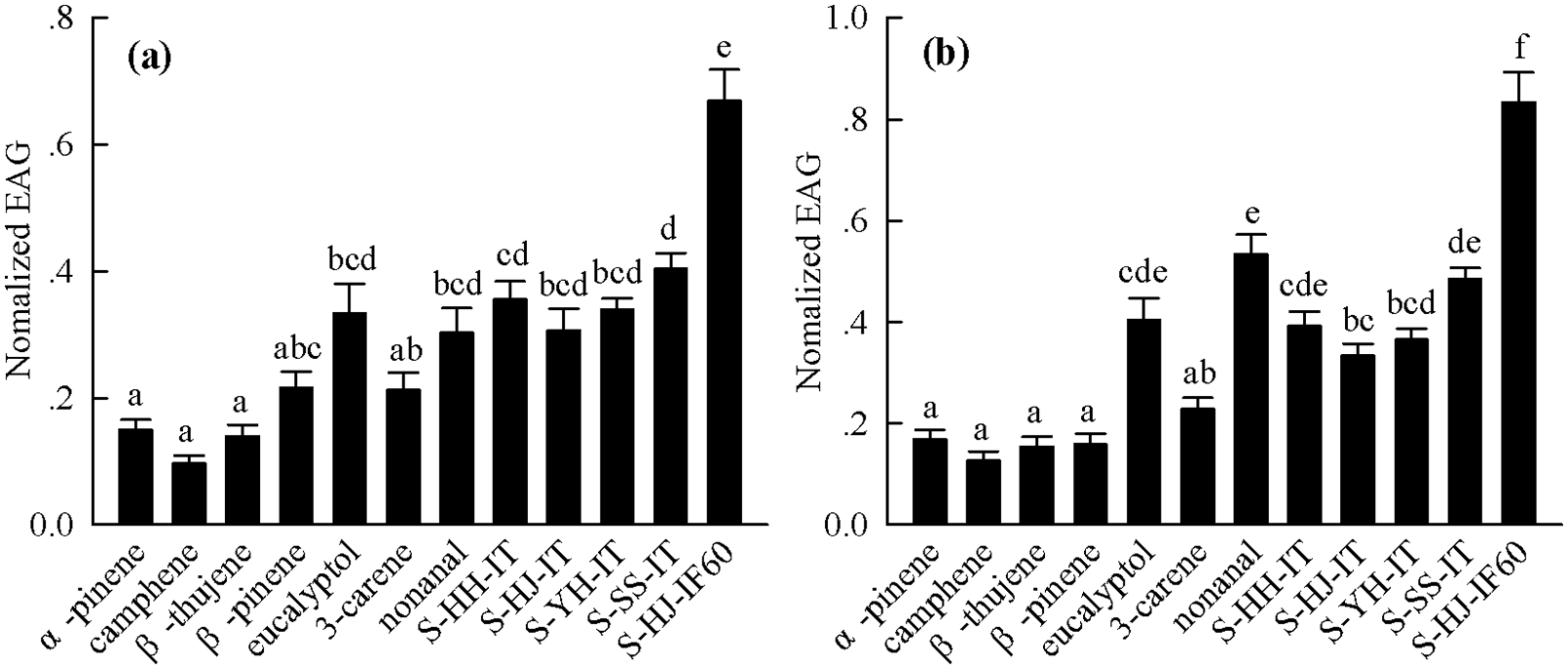
EAG response profiles of female *Conogethes punctiferalis* to chestnut compounds. (a) EAG responses of virgin females to chestnut compounds, (b) EAG responses of mated females to chestnut compounds, S-HH-IT = simulated blend mimicking the emission of intact Huaihuang fruits, S-HJ-IT = simulated blend mimicking the emission of intact Huaijiu fruits, S-YH-IT = simulated blend mimicking the emission of intact Yanhong fruits, S-SS-IT = simulated blend mimicking the emission of intact Shisheng fruits, S-HJ-IF60 = simulated blend mimicking the emission of Huaijiu fruits infested by *Conogethes punctiferalis* for 60 h. Bars represent mean ± SE (*n* = 15).

Electroantennogram (EAG) responses of virgin and mated *C*. *punctiferalis* females to the single compounds and the simulated blends (Table 1) were also tested. All seven test compounds, *α*-pinene, camphene, *β*-thujene, *β*-pinene, eucalyptol, 3-carene, and nonanal, triggered EAG responses in *C*. *punctiferalis* (Fig 6a and 6b). Additionally, all simulated mixture blends, S-HH-IT, S-HJ-IT, S-YH-IT, S-SS-IT, and S-HJ-IF60, triggered EAG responses in virgin and mated females (Fig 6a and 6b).

## Discussion

In lepidopteran insects, host selection by adult females is crucial for the fitness of the caterpillars [33]. Although *C*. *punctiferalis* is able to feed on a variety of plant species, our previous study demonstrated that the caterpillars’ performance varies from host to host [29]. Therefore, it seems likely that *C*. *punctiferalis* adults exhibit preference for some host plants based on their underlying effects on caterpillars’ performance. In the present study, we demonstrated that adult female *C*. *punctiferalis* preferred to oviposit on the chestnut cultivar Huaijiu over the cultivars Huaihuang, Yanhong, and Shisheng (Fig 1b). Furthermore, in the field Huaijiu cultivar was subjected to higher infestation rates than the other three chestnut cultivars planted in the same orchard (Fig 1a), confirming a preference of *C*. *punctiferalis* for Huaijiu.

The preference-performance hypothesis has been proved in many phytophagous insects [10]. However, in the case of lepidopteran species over half of studies conducted to investigate the relationship between oviposition preference and caterpillar performance find no correlation [11]. This arouses our interest to explore whether or not the preference of *C*. *punctiferalis* females for Huaijiu correlated with the performance of the caterpillars. The physiological adaptation of caterpillars to different host plants can be described by calculating different parameters that characterize the efficiency of host use [34]. In the present study, we found that the development, survival and reproduction of *C*. *punctiferalis* varied among different chestnut cultivars (Figs 2 and 3), demonstrating differential suitability among cultivars. As shorter development duration and higher fecundity of insects on a host indicate greater suitability of the host plant [35]; therefore, Huaijiu was the most suitable host for *C*. *punctiferalis*. Linking the host preference of adult females with the performance of the caterpillars, we conclude that there is a positive relationship between the host preference and the performance of *C*. *punctiferalis*. This relationship supports the hypothesis that adult females of *C*. *punctiferalis* can preferentially select more suitable host plants for oviposition and consequently provide benefit to their offspring. However, because we only assessed the preference-performance relationship by asking whether females ranked their host plants based on their food quality for offspring, the results might not reflect the outcome in a more complex ecological context. A variety of factors, such as microclimatic conditions, mutualists, competitors and/or natural enemies in ecological contexts, could make significant contributions to the host selection behavior of adult females and to the performance of their offspring [10]. Future work studying the interaction between *C*. *punctiferalis* and chestnuts will address these issues.

Plants produce chemical cues, both volatile and non-volatile, that can be used by insects as information for searching suitable host plants for feeding or oviposition. Many studies with laboratory bioassays have demonstrated that plant volatiles play important roles in the host recognition and location behavior of lepidopteran insects [22,36]. In the present study, mixture blends mimicking the volatiles emitted from the four chestnut cultivars were attractive to both virgin and mated *C*. *punctiferalis* females (Fig 5), indicating that the *C*. *punctiferalis* females use plant volatiles to recognize and locate their host plants. Four ubiquitous plant terpenoids, *α*-pinene, camphene, *β*-thujene and *β*-pinene, were commonly emitted by chestnut fruits (Table 2), of which *α*-pinene, camphene and *β*-pinene triggered significant EAG responses in *C*. *punctiferalis* and showed attractiveness to both virgin and mated females. It was therefore speculated that these compounds, or a blend of them, may be used by *C*. *punctiferalis* as cues for host recognition and location because many ubiquitous compounds, when combined in specific proportions, can trigger host location behaviors in insects [22,23]. Modulation in the ratios of ubiquitously essential compounds in grape *Vitis riparia* was sufficient to affect the host recognition behavior of female grape berry moth *Paralobesia viteana* [24]. Quantitative differences in the emission amount and relative proportion of *α*-pinene, camphene, *β*-thujene and *β*-pinene were observed among the four chestnut cultivars (Table 2). However, regardless of the difference in concentration and proportion of the four terpenoids, all simulated mixture blends showed attractiveness to *C*. *punctiferalis* females (Fig 5), indicating an olfactory plasticity of *C*. *punctiferalis* in locating hosts within distinct concentrations or proportions of volatile blend constituents. The plasticity was suggested to play important roles in host recognition and acceptance of insect herbivores [37–41]. A certain degree of olfactory plasticity has been evolved in some insect herbivores for enhancing the location of their hosts [42]. Grape berry moth *Paralobesia viteana*, for example, has the plasticity to orient to an odor source regardless of the variation in the ratio or the concentration of the mixture [24]. Female grapevine moth, *Lobesia botrana*, could be attracted to both specific as well as common plant odor cues, indicating plasticity in host recognition by plastically responding to partial odor blends of host plants [43]. Our results also suggest a plastic recognition system in the polyphagous herbivore *C*. *punctiferalis*, and this plasticity may be an adaptation to the changes in ratio and concentration of volatiles emitted by different host plants. Thus, the plasticity might, in combination with host plant diversity and larval suitability, be very important for the forming of the interactions between yellow peach moth and chestnut cultivars.

Plant volatiles can also be induced by feeding or oviposition of herbivores [18,36,37,44]. Qualitative and quantitative changes of volatile emissions in response to attack by herbivores have been intensively investigated [45,46]. Herbivore-induced plant volatiles may be used as cues for host recognition and selection as infested plants are usually avoided by female moths to minimize competition among their relatively immobile progeny [47]. The release of caterpillar-feeding-induced chestnut volatiles and their potential effects on host recognition by *C*. *punctiferalis* were investigated in the present study, using Huaijiu as a representative. Aside from the commonly emitted *α*-pinene, camphene, *β*-thujene and *β*-pinene, another five volatiles including eucalyptol, *β*-ocimene, 3-carene, nonanal and (*E*)-2-butenoic acid, 2-(methylene-cyclopropyl)prop-2-yl ester were specifically emitted in response to infestation by *C*. *punctiferalis* (Fig 4b and Table 3). In addition, the emission of *C*. *punctiferalis*-induced volatiles was time dependent, with *β*-ocimene and (*E*)-2-butenoic acid, 2-(methylene-cyclopropyl)prop-2-yl ester emitted from early stage of infestation (12 h and 36 h), whereas eucalyptol, 3-carene, and nonanal were emitted at the late stage of infestation (60 h). Behavioral responses of *C*. *punctiferalis* females to herbivore-induced volatiles were tested using artificially prepared blends simulating the emissions of intact (S-HJ-IT) and *C*. *punctiferalis* infested (S-HJ-IF60) Huaijiu fruits. S-HJ-IT showed significant attraction to both virgin and mated females, whereas S-HJ-IF60 showed repellence (Fig 5). The most obvious difference between S-HJ-IF60 and S-HJ-IT was the presence of nonanal (Table 3), a compound that showed strong repellence to *C*. *punctiferalis*. It is therefore suggested that nonanal plays a critical role in mediating the recognition of intact and infested fruits.

## Conclusion

In this study, we investigated the preference and performance of polyphagous *C*. *punctiferalis* on four chestnut cultivars, Huaihuang, Huaijiu, Yanhong, and Shisheng. We found that in field Huaijiu had higher infestation rate than the other three chestnut cultivars and in laboratory *C*. *punctiferalis* females chose to lay more eggs on Huaijiu than on the other three chestnut cultivars. Rearing experiments showed that the *C*. *punctiferalis* caterpillars fed on Huaijiu had better fitness than those fed on the other three chestnut cultivars. Taken together, these results demonstrate a correlation between adult preference and caterpillar performance in *C*. *punctiferalis*. However, tests of behavioral responses of *C*. *punctiferalis* to chestnut volatiles demonstrated that the females could plastically sense and respond to the emissions of the four chestnut cultivars. Simulated blends mimicking the volatiles emitted from the four chestnut cultivars all showed attractiveness to *C*. *punctiferalis* females. The simulated blend mimicking the volatiles emitted from *C*. *punctiferalis* infested Huaijiu fruits elicited a repellent response. Seven chestnut volatiles, *α*-pinene, camphene, *β*-thujene, *β*-pinene, eucalyptol, 3-carene, and nonanal, all triggered significant EAG responses in *C*. *punctiferalis*, of which *α*-pinene, camphene and *β*-pinene showed attractiveness while nonanal showed repellence to both virgin and mated females. Altogether, these results demonstrate the effects of chestnut cultivars on the performance and fitness of *C*. *punctiferalis*. Even though there is a preference-performance relationship between *C*. *punctiferalis* adults and their offspring, there is olfactory plasticity in the plant-volatile-mediated host recognition of female *C*. *punctiferalis*.

## Supporting Information

S1 Fig Setup used for oviposition selection test. (PDF)

S2 Fig Setup used for chestnut volatiles collection. (PDF)

S3 Fig Process of artificially introducing *Conogethes punctiferalis* onto chestnut fruits. (a) *Conogethes punctiferalis* caterpillar was introduced using a Chinese brush pen onto a chestnut bur, (b) and (c) The caterpillar was boring into the bur, (d) The caterpillar had completely bored into the bur, (e) and (f) The caterpillar successfully settled down and started to feed in the bur.
(PDF)
