# Supplementary material for: Host Preference and Performance of the Yellow Peach Moth (Conogethes punctiferalis) on Chestnut Cultivars
Source: PLoS One. 2016 Jun 21;11(6):e0157609. doi: 10.1371/journal.pone.0157609 (PMC4915626; doi:10.1371/journal.pone.0157609)
Supplement: S1 Fig — (PDF) [file pone.0157609.s001.pdf]

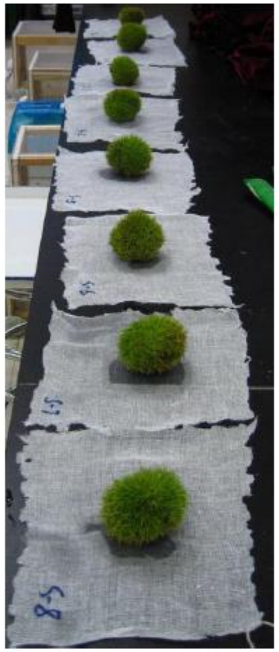

Preparation of chestnut burs for oviposition selection

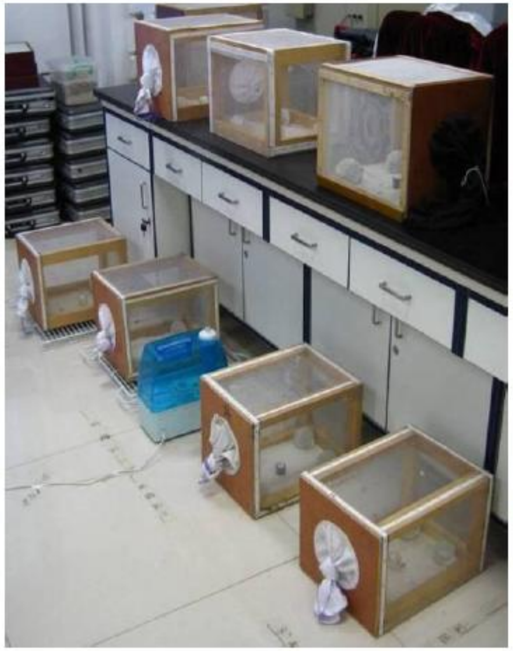

Wood-frame cage (35 cm×27cm ×25 cm) used for oviposition selection test

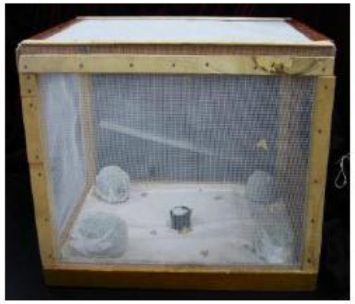

Profile view of the test cage

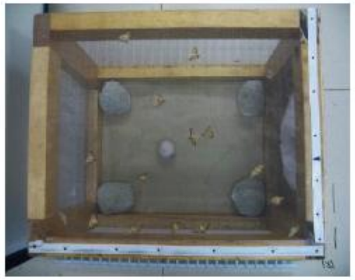

Plan view of the test cage

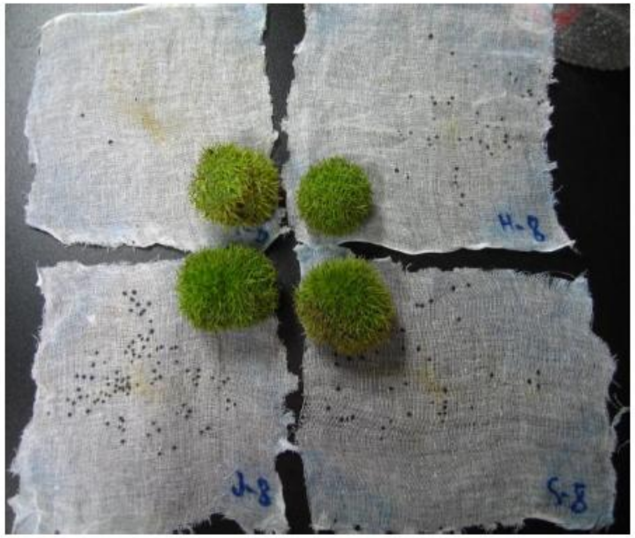

Clothes with *Conogethes punctiferalis* eggs (marked in black)
