# Supplementary figures and images for: Host Preference and Performance of the Yellow Peach Moth (Conogethes punctiferalis) on Chestnut Cultivars
Source: PLoS One. 2016 Jun 21;11(6):e0157609. doi: 10.1371/journal.pone.0157609 (PMC4915626; doi:10.1371/journal.pone.0157609)

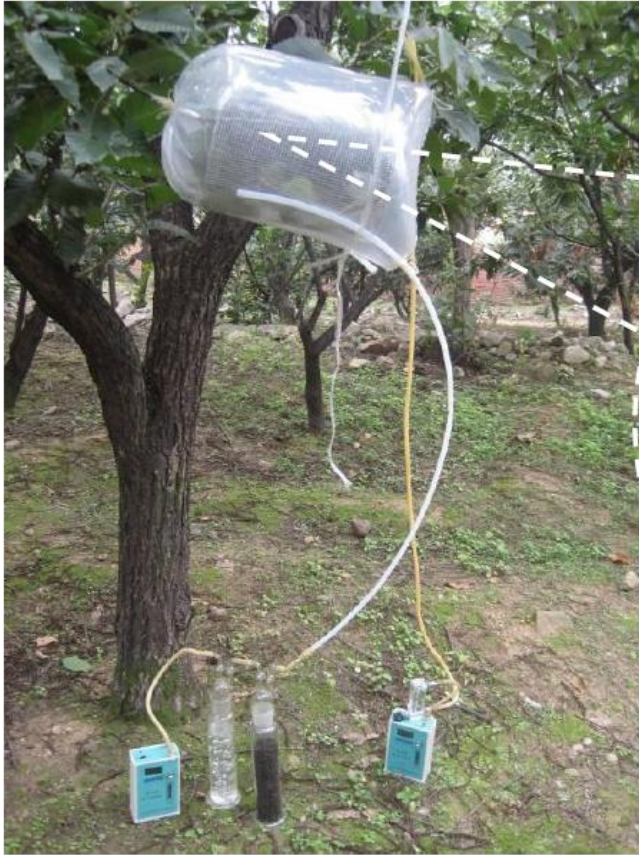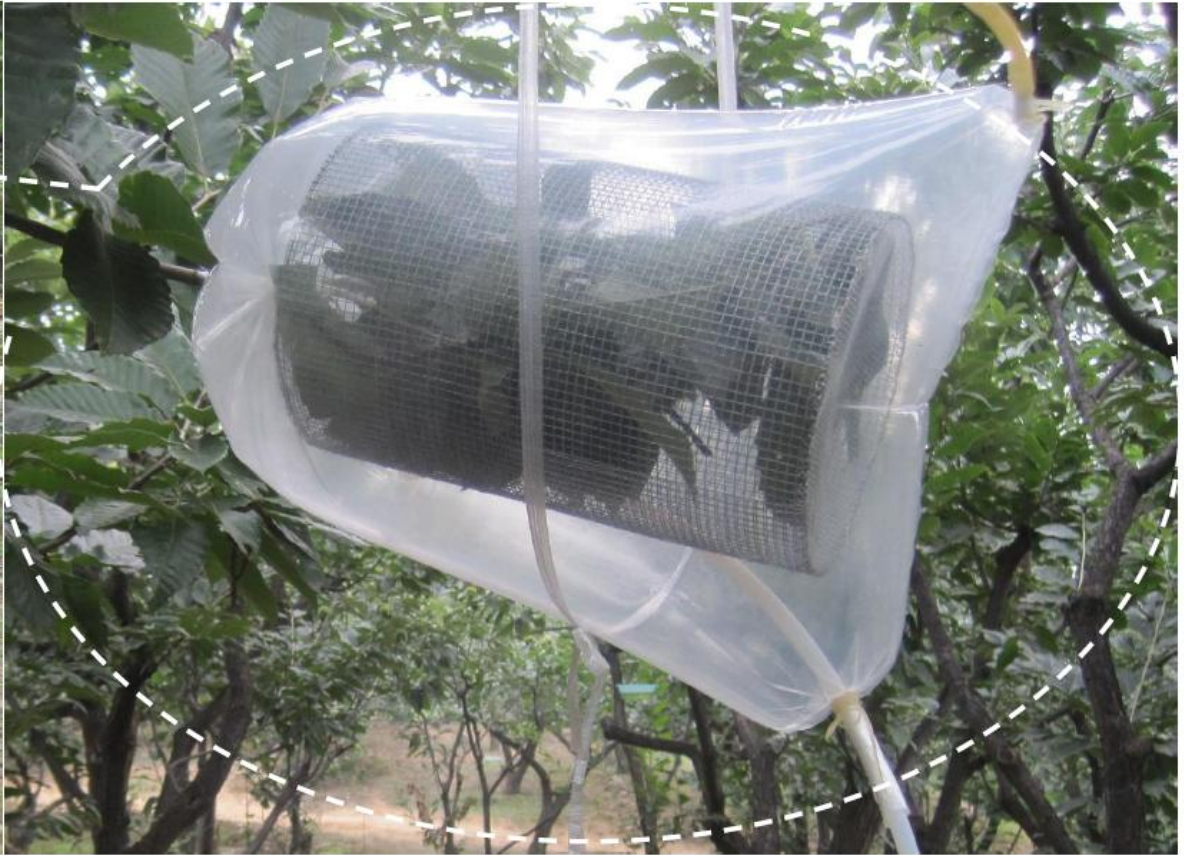

Supplement: S2 Fig — (PDF) [file pone.0157609.s002.pdf]

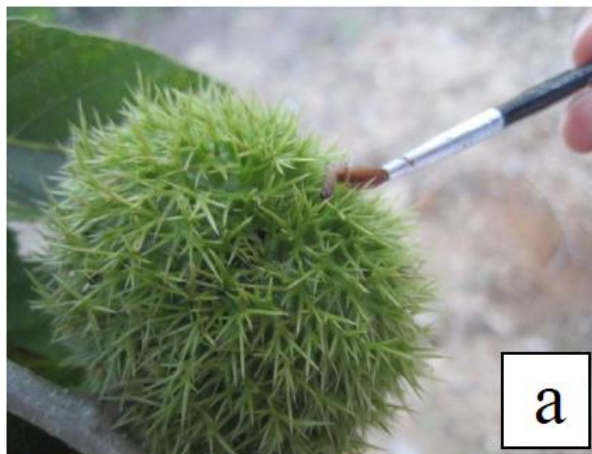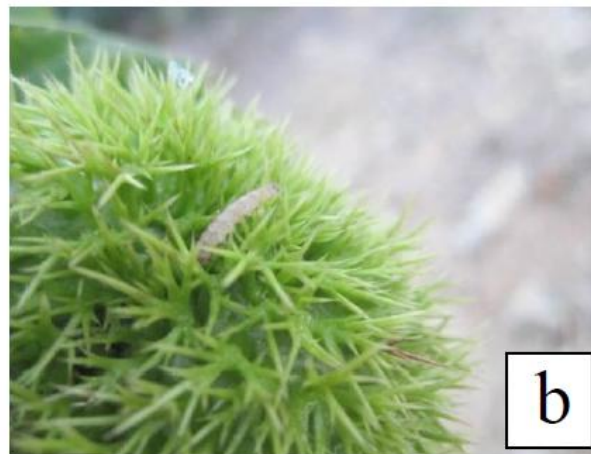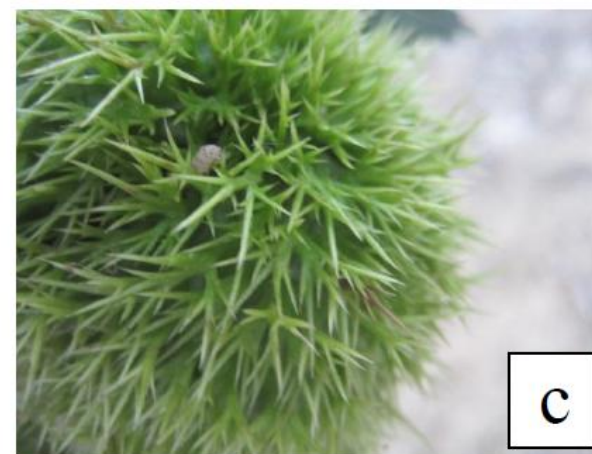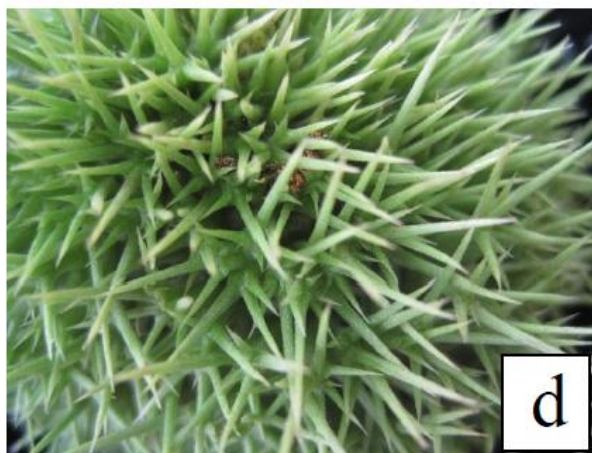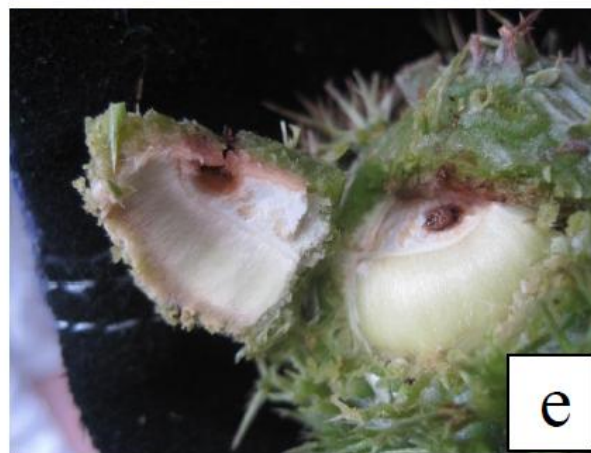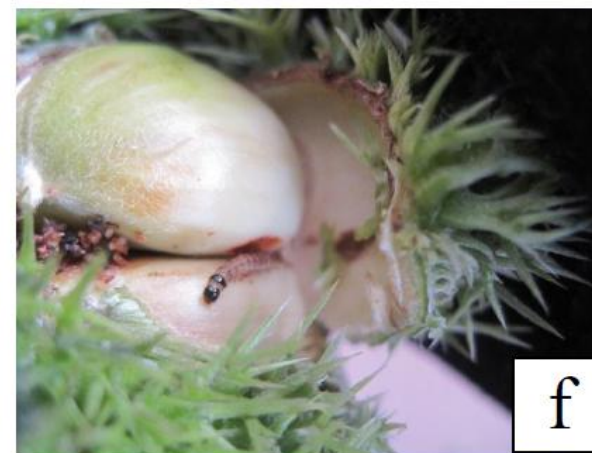

Supplement: S3 Fig — (a) Conogethes punctiferalis caterpillar was introduced using a Chinese brush pen onto a chestnut bur, (b) and (c) The caterpillar was boring into the bur, (d) The caterpillar had completely bored into the bur, (e) and (f) The caterpillar successfully settled down and started to feed in the bur. (PDF) [file pone.0157609.s003.pdf]
